# Sulfur Enhancement for the Improvement of Castor Bean Growth and Yield, and Sustainable Biodiesel Production

**DOI:** 10.3389/fpls.2022.905738

**Published:** 2022-07-04

**Authors:** Ahmed Mukhtar, Masood Iqbal Awan, Sana Sadaf, Athar Mahmood, Talha Javed, Adnan Noor Shah, Rubab Shabbir, Saqer S. Alotaibi, Anis Ali Shah, Robert Adamski, Dorota Siuta

**Affiliations:** ^1^Department of Agronomy, University of Agriculture Faisalabad, Faisalabad, Pakistan; ^2^Department of Agronomy, Sub-Campus Depalpur, Okara, University of Agriculture Faisalabad, Faisalabad, Pakistan; ^3^Punjab Bioenergy Institute, University of Agriculture, Faisalabad, Pakistan; ^4^College of Agriculture, Fujian Agriculture and Forestry University, Fuzhou, China; ^5^Department of Agricultural Engineering, Khwaja Fareed University of Engineering and Information Technology, Rahim Yar Khan, Pakistan; ^6^Seed Science and Technology, University of Agriculture Faisalabad, Faisalabad, Pakistan; ^7^Department of Biotechnology, College of Science, Taif University, Taif, Saudi Arabia; ^8^Department of Botany, University of Education, Lahore, Pakistan; ^9^Faculty of Process and Environmental Engineering, Łódź University of Technology, Łódź, Poland

**Keywords:** castor bean, biodiesel, sulfur, sustainability, non-edible oil

## Abstract

Due to limited conventional energy sources, there is a need to find substitute non-conventional sources of energy to meet the societal demands on a sustainable basis. Crude oil and edible oil remain major import items in Pakistan, the deficit of which can be compensated by using biomass, preferably inedible oilseeds. Therefore, the current study evaluated the role of sulfur (S) fertilization for improving yield (seed and oil) and biodiesel value of castor bean, a potential inedible crop with minimum input requirements. For this purpose, a combined approach of field experimentation and laboratory analysis was conducted to explore the potential of two castor bean cultivars (DS-30 and NIAB Gold) against four S supply rates, namely, 0, 20, 40, and 60 kg S ha^–1^, in terms of growth, phenology, and yield parameters. Subsequently, the obtained seed samples were analyzed for biodiesel-related parameters in the Bio-analytical Chemistry lab, Punjab Bio-energy Institute, Faisalabad. The incremental S rates increased the seed yield for both cultivars, and the highest yield was recorded at 60 kg S ha^–1^ for NIAB Gold. For NIAB Gold, the oil content increased by 7% with S fertilization at 60 kg ha^–1^, and for DS-30, the oil content increased by 6% at 60 kg ha^–1^. As with incremental S fertilization, the oil yield increased on a hectare basis, and the quantity of biodiesel produced also increased. Importantly, the tested quality parameters of biodiesel, except biodiesel viscosity, were in the ASTM standard range. Overall, it has been concluded that castor bean is a promising and sustainable option for producing biodiesel as it is non-competitive to food crops and requires little input.

## Introduction

Environmental pollution caused by fossil fuel burning, high economic cost of importing petroleum products, and their supply chain disruptions are the prime concerns of this century (Azam and Shafique, 2017). Fossil fuels are non-renewable energy sources, and their extensive utilization is projected to exhaust their reservoirs in near future. In addition, the changing dynamics of industrial functioning has necessitated harnessing eco-friendly petroleum alternatives which are not only economical but also sustainable in the longer run (Chen et al., 2012; Javed et al., 2021; Mahmood et al., 2021). The biodiesel prepared from non-staple crops holds potential to bring revolution in the petroleum sector along with putting a halt to environmental degradation (Carvalho et al., 2020; Muanruksa et al., 2020).

Biodiesel can be produced from edible or inedible crops; however, the utilization of food crops for this purpose has been discouraged due to food security concerns (Toldrá-Reig et al., 2020). Castor bean (*Ricinus communis*) belonging to family Euphorbiaceae has been found to be a promising crop for biodiesel production (Novaes et al., 2020). It can produce stable yields even on marginal lands for being tolerant to water stress, low soil fertility, high soil pH, and arid conditions (Chuah et al., 2016). Exploring the biodiesel potential of castor bean has received less attention from the researchers and policymakers, and the prevalent scenario of severe environmental degradation has made it necessary to explore it as a sustainable source of biofuel production (Aguado-Deblas et al., 2020; Arslan et al., 2020; Osorio-González et al., 2020).

Commercial production of castor bean requires enhancing both the profitability and sustainability of castor-based cropping systems. Optimal fertilization is the key to realizing the full yield potential of castor bean. The secondary macro-nutrient sulfur (S) is particularly essential for increasing the seed yield, oil content, and oil yield and hence biodiesel production (Kamran et al., 2019; Zaheer et al., 2020). Mostly arable agricultural soils hold a very small quantity of inorganic S, which is bound in nature and is not readily available for plants (Hashem et al., 2020). S is found in two important amino acids, methionine and cysteine, and is part of several coenzymes or vitamins that are vital for plant metabolism (Chotchutima et al., 2016; Saleem et al., 2020a). Sharma and Gupta (2003) performed a study to observe the impact of S on the plant physiology and yield of castor bean and concluded that S application rates, namely, 20, 40, and 60 kg S ha^–1^ enhanced the yield over control in castor. The effect of S fertilization on growth and yield of castor bean has been studied in many previous studies (Ahmad et al., 2007; Ren et al., 2017; Sutton et al., 2019). In addition to the potential multipurpose uses of castor (Ramanjaneyulu et al., 2013; Table 1), the due considerations of biodiesel production from castor can mitigate our energy crisis and greenhouse gas emissions.

**TABLE 1:** Various uses of castor bean and its products.

Sr. No.	Uses	Method of use	References
1	Organic nutrient source	Castor bean seed cake contains 6.6% N, 2.6% P_2_O_5_ and 1.2% K_2_O. Seed cake applied to agricultural fields	Ramanjaneyulu et al., 2013
2	Biogas generation	Castor bean seed cake used in biogas generation	Lingaiah and Rajasekaran, 1986
3	Ericulture	Castor bean plant leaves used as Eri silkworms food.	Narayanamma, 2018
4	Pest control	Castor extract is useful against adults of different insects, storage pests in coffee, soil nematodes, insects and fungal casual agents.	Zahir et al., 2010
5	Lubrication	Castor oil maintains higher viscosity and is widely used as a lubricant in jet, diesel and race engines.	Ramanjaneyulu et al., 2017
6	Biodiesel and Bioethanol	The cultivation of castor has encouraged for biodiesel and bioethanol production in Brazil	Oliveira et al., 2008
7	Soil remediation	Castor is suitable for remediation of crude oil contaminated soil	Vwioko et al., 2006
9	Phytoremediation	Castor bean has the potential to tolerate and accumulate heavy metals like Cd	Bauddh and Singh, 2012
10	Removal of heavy metals from water	Castor leaf powder acts as a green adsorbent for the removal of heavy metals from aqueous solutions	Martins et al., 2013
12	Coating and paints	Coatings and paints, paint or furniture oil applications	Osorio-González et al., 2020
13	Polymer materials	Castor oil and its derivatives can be used in the synthesis of renewable monomers and polymers	Mutlu and Meier, 2010
14	Soaps, waxes, and greases	Soap for washing, grease for nuts and bolts	Burt and Mealy, 1942
15	Pharmacological and medicinal	often used as drug delivery vehicle for very non-polar drugs such as the anti-cancer drugs paclitaxel and docetaxel. Ricinoleic acid in caster is used for the treatment of gastrointestinal tract e.g., use as anti-diarrhea activity.	Ramanjaneyulu et al., 2013; Arslan et al., 2020
16	Chemical Industry Uses	ricinoleic acid (RA) is content of caster, which is used in the chemical industry	Ramanjaneyulu et al., 2013

Economizing farmer’s cost of production and enhancing sustainability are a major focus of policy guidelines. Against this background, selection of potential cultivars and developing site-specific sound agronomic management practices under Pakistani conditions for biodiesel crops like castor bean can raise the farmers’ socio-economic status. Keeping in view the importance of this crop, the Nuclear Institute of Agriculture and Biology (NIAB, Pakistan) developed two promising castor bean cultivars, namely, DS-30 and NIAB Gold, which are high yielding with a high seed oil content (range 50–60%) and early maturing (3 months). Due to these attributes, these varieties can be integrated into our existing cropping systems. Cheema et al. (2013) performed trials with different cultivars of castor bean and argued that cv. DS-30 is the most suited for medium- and low-rainfall ecologies, while PR-101 produced better yield under low-rainfall conditions. Therefore, the present study aims to explore the research queries regarding the possibility of growing castor for biodiesel purposes and whether S nutrition can help improve yield and biodiesel value of castor bean. The findings from the present study will add to the knowledge of researcher/readers about the comparison of two caster bean cultivars for its seeds, oil, biodiesel yield on per hectare basis, and its quality according to the standards of the (i) American Society for Testing and Materials (ASTM) and (ii) quantification of an optimum S dose for improving yield and biodiesel value of castor bean cultivars.

## Materials and Methods

### Field Experimental Site and Layout

**FIGURE 1:**
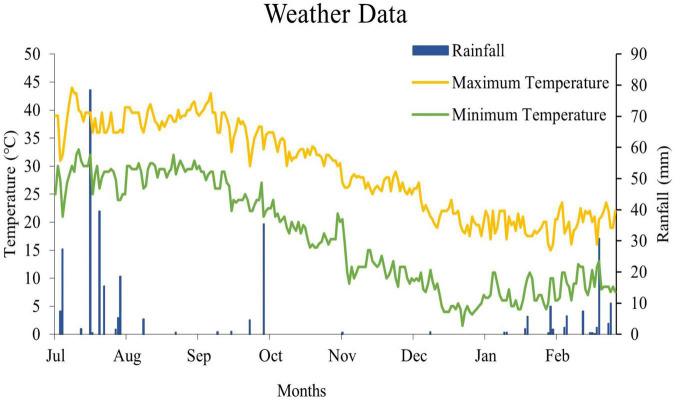
Temperature and rainfall data of the growing season at field experimental site.

In order to achieve the aforementioned objectives, a dedicated field experiment was carried out in 2018 at the Post-graduate Agricultural Research Station (PARS) farm of the University of Agriculture Faisalabad, Pakistan (31.38° N, 73.01° E). The climate of this region is semiarid to subtropical. The daily weather data on maximum and minimum air temperature and rainfall, collected from the meteorological observatory of the UAF, for the castor-growing season are shown in Figure 1.

Data on soil physical and chemical characteristics, analyzed from the pre-sowing unfertilized soil samples, are tabulated in Table 2. Our field experiment tested two castor bean cultivars (DS-30 and NIAB-Gold) in response to four sulfur (S) supply rates (S_1_: control, S_2_: 20 kg S ha^–1^, S_3_: 40 kg S ha^–1^, S_4_: 60 kg S ha^–1^). Experimental design was randomized complete block design (RCBD) in split-plot arrangement with four replications. Factor A was castor bean varieties in main plots, and factor B was S rates in subplots. The net plot size was 4 m × 3 m, and the gross plot size was 5 m × 3 m.

**TABLE 2:** Physio-chemical analyses of soil before sowing.

	Determination	Value
Structural analysis	Texture class	Sandy loam
Chemical analysis	Nitrogen	0.05%
	Phosphorus	8.4 ppm
	Potassium	137 ppm
	Sulfur	7.05 ppm
	Organic matter	0.6%
	E.C	1.4 dS m^–1^
	pH	7.6

### Crop Husbandry

Preparation of the experimental site started using the “Daab method.” The field was irrigated to allow weed seeds to germinate. After germination, the field was cultivated to incorporate weeds. After 5 days, field was re-irrigated to allow the remaining weed seeds to germinate. The emerged weeds were incorporated into the soil by plowing. Then, the fine seedbed was prepared by two to three ploughings, followed by light planking. Seeds were sown at 16 kg ha^–1^ on 18 August 2018 using a dibbler to maintain a 30-cm plant-to-plant spacing and a 100-cm row-to-row spacing. Recommended rates of fertilizers at 110 kg N applied by urea (46% N) and DAP (18% N), 25 kg ha^–1^ P2O5 supplied by DAP, and 45 kg ha^–1^ K_2_O supplied by MOP (60% K) were applied. In respective plots (or treatments), S was applied as gypsum (CaSO_4_.2H_2_O) with first irrigation. Totally, three irrigations of 4 acre-inch were applied according to crop requirements on the 2nd week after germination, at flowering and at seed filling. The crops were harvested at maturity.

Due to indeterminant nature of crop, a total of three pickings were done: first picking on 10th January 2019, second on 05 February 2019, and third on 1st March for NIAB Gold, and first picking on 20th February 2019, second on 15 March 2019, and third on 25 April for DS-30.

### Field Measurements

Agronomic data on crop parameters such as plant height, leaves per plant, spike length, branches per plant, spikes per plant, capsules per plant, days to 50% flowering, and crop duration, hundred seed weight, and seed yield were recorded following standard procedures. Based on these parameters, we also calculated the seed oil content (%), oil yield per hectare, and biodiesel yield. We used five randomly selected plants to record different agronomic traits from each treatment. The plant height of castor beans was measured using a measuring scale from the tip of the roots to the shoot tip and number of branches were measured straightway.

### Laboratory Analysis: Oil Yield and Content Determination

We analyzed biodiesel-related parameters in the Bio-analytical Chemistry lab, Punjab Bio-energy Institute (PBI), University of Agriculture Faisalabad (UAF) (31.38°N, 73.01°E). The oil content was determined by using an electric Soxhlet apparatus. Briefly, 200 g seeds were oven-dried, followed by grounding and filling in a thimble. An n-hexane solvent was used to extract the oil. The mixture of n-hexane and oil was separated from the Soxhlet. The oil from n-hexane was separated by a rotary evaporator and expressed in kilograms per hectare.

### Synthesis of Biodiesel

Castor bean seed extracted refined oil was transformed into fatty acid methyl esters (biodiesel). For the transesterification process, KOH was mixed in measured quantity (methanol:oil 5:1) of methanol, and then oil was added to this solution. This mixture was heated on a hot plate at a temperature of 65°C for 3 hours. A magnetic stirrer was used for stirring at 400 rpm. Stirring is required for supporting the reaction mixture for the synthesis of fatty acid methyl esters (FAMEs). After completing the transesterification process, the mixture of reaction was kept in a separating funnel and left for 36 hours to settle down. In the lower portion of the funnel, glycerine was settled. The upper layer comprises FAME and collected separately.

### Biodiesel Quantitative and Qualitative Attributes

Biodiesel yield was determined on the basis of oil amount by using the following formulas:


BiodieselYield(%)=

mL⁢of⁢biodiesel⁢produced/mL⁢of⁢oil⁢used⁢in⁢reaction

×100

Biodiesel yield ha-1 calculated on hectare and kilogram basis by using below formula.


BiodieselyieldLha=-1Oilyieldha×-1Biodieselyield(%)

#### Determination of Acid Value

Free fatty acids in oil and biodiesel were estimated by the determination of its acid value. For the determination of acid value, a measured amount of biodiesel was dissolved into ethanol. Then, phenolphthalein indicator was added into the reaction solution. Only one to two drops of phenolphthalein indicator were added to solution. This mixture was then titrated with 0.1 N potassium hydro-oxide (KOH) until the appearance of pink color in solution.

Free fatty acid concentration in biodiesel was calculated by using the following formula:


Free⁢fatty⁢acid=V×N×28.2/W

Here, V is the volume and N is the normality of KOH used for titration, and W is the weight of biodiesel used. Then biodiesel acid value was measured with the help of the following formula:


Acidvalue=%FFA×1.989

#### Determination of Calorific Value

An Oxygen Bomb Calorimeter (model: DSHY-1A + ZhauhaiDshing) was used for the measurement of castor bean biodiesel calorific value.

#### Determination of Iodine Value

Iodine value was used to measure the amount of unsaturated fatty acid. For this purpose, 1 g of biodiesel sample was put into CCI4 solution. After that 25 mL of wijs reagent was added in this solution, and the flask containing this solution was kept in a dark place for 30 min. Potassium iodide (15%) solution was prepared and added into reaction solution, followed by adding 100 mL distilled water. Starch solution (2–3) drops as the indicator of titration were added, and titration was carried out against 0.1 N sodium thiosulfate (Na2S2O3.5H2O) till the yellow color of solution disappeared. The solution yellow color was an indicator of iodine presence. The same procedure was applied to the blank solution.

Iodine value was estimated by using the following formula:


Iodine⁢value=(B-S)×N×12.69/W

Here, B represents the amount 0.1 N sodium thiosulfate (mL) required by the blank sample, S presents 0.1 N sodium thiosulfate (mL) required by the biodiesel sample, N for normality of sodium thiosulfate, and W stands for the biodiesel weight (g).

#### Determination of Saponification Value

For the determining the ability of biodiesel to make soap, the saponification value of castor bean biodiesel was estimated by titration of biodiesel with 0.5 N ethanolic KOH. The reaction solution (1 g biodiesel and 20 mL of 0.5 N ethanolic KOH) was taken in a flask and heated with a hot plate at 400°C with the support of a water condenser. Solution was heated and stirred till the appearance of a clear solution. Clear solution tells about the completion of this reaction. The flask was removed from the hot plate and kept at room temperature. Phenolphthalein solution (2–3) drops as a titration indicator were added to the reaction solution. This reaction mixture was titrated against 0.5 N HCI till disappearance of pink color. Blank solution was taken following the same practices and titrated with 0.5 N HCl solution.

Saponification value was measured using the following formula:


Saponification⁢value⁢(SV)=(B-S)×N×56.1/W

Here, B stands for the quantity 0.5 N HCl (mL) for blank reading, S indicates the amount (mL) of HCl used for the biodiesel sample, N is the normality of HCl, and W represents the biodiesel weight (g).

#### Determination of Cetane Number

The cetane number of castor bean synthesized biodiesel was measured using the following formula:


Cetane⁢Number=46.3+ 5458/SV-0.225×IV

Saponification value signifies the saponification value and IV indicates the iodine value of synthesized biodiesel as calculated using the following formulas:

#### Determination of Viscosity

A viscometer (Model: DV2T, Brookfield) was used to measure the viscosity of biodiesel.

#### Pure Point and Cloud Point

**FIGURE 2:**
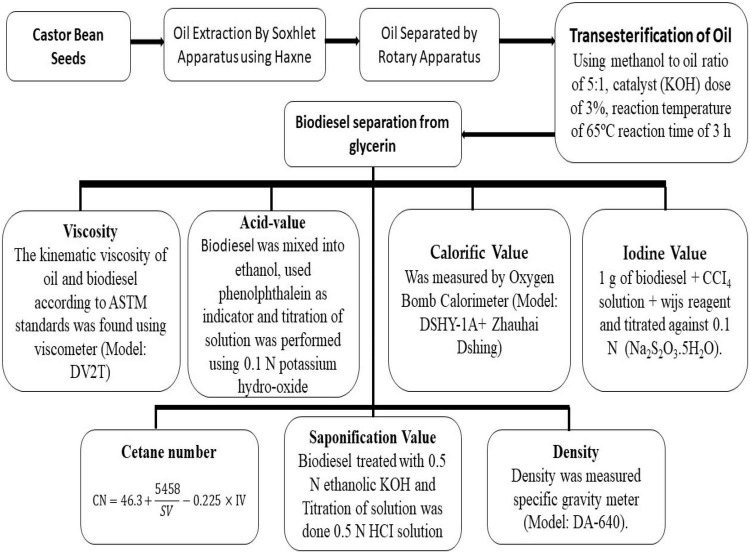
A proposed schematic representation of complete study design and sustainable biodiesel production from castor bean.

Biodiesel cloud and pour point were determined by placing the sample into a refrigerator. The sample was taken into a glass test tube, and a thermometer was inserted into it. The clear biodiesel sample turns cloudy, and this temperature was noted as the cloud point. Then again, the sample was placed in the refrigerator, and after some time, the biodiesel movement slowed down due to decreasing temperature this point was taken as the pure point. A proposed schematic representation of the complete study design and sustainable biodiesel production from castor bean has been given in Figure 2.

### Statistical Analysis

Fisher’s analysis of variance and LSD test at 5% level of probability were employed to compare the treatment means (Steel, 1997).

## Results and Discussion

### Morphology and Yield Attributes

Plant height is a crucial indicator of yield, and it is a function of genetic composition of the crop. Both cultivars and S application significantly (*p* ≤ 0.01) affected the plant height of castor bean (Table 3). Plant height increased with increasing S dose in both cultivars. NIAB-Gold produced taller plants and higher number of branches and spikes plant^–1^ at 60 kg S ha^–1^. The plant height of DS-30 was 7% higher than that of NIAB Gold, which might be due to genetic variability and crop duration (Table 4). In both cultivars, the number of leaves per plant was significant at 60 kg ha^–1^. Mean leaves per plant (96) in DS-30 was 47% higher than that of NIAB Gold (data not given), which shows that DS-30 had a more effective photosynthetic area.

**TABLE 3:** ANOVA sources, *F*-values, and levels of statistical significance in plant morphology and biomass yield.

Source	DF	PH	SL	BPP	SPP	SDW	DTF	CPP	HSW	DTM	SY	OC	OY	BDY	BDY
		(cm)	(cm)			(g)	Days		(g)	Days	Mg ha^–1^	(%)	kg ha^–1^	L kg^–1^	L ha^–1^
V	1	2315.34**	11.25^NS^	22.16*	7.70^NS^	1.35^NS^	50.84*	0.00^NS^	0.41^NS^	50.73*	3.38^NS^	2.73^NS^	20.48*	230.82**	87.89*
S	3	103.49**	2.08^NS^	701.83**	381.93**	38.57**	39.32**	2.99*	8.10**	39.38**	3.49*	36.91**	18.69**	110.78**	42.11**
V X S	3	12.98**	0.49^NS^	1.33^NS^	5.72*	3.40^NS^	4.04*	0.79^NS^	0.24^NS^	4.05*	0.02^NS^	2.37^NS^	1.40^NS^	5.11*	0.53^NS^

DF, degree of freedom; V, variety; S, sulfur; PH, plant height; SL, spike length; BPP, branches per plant; SPP, spike per plant; SDW, spike dry weight; DTF, days taken to 50% flowering; CPP, capsule per plant; HSW, hundred seed weight; SY, seed yield; DTM, days taken to maturity; OC, oil content; OY, oil yield; BDY, biodiesel yield. NS, *, and ** indicate not significant, and significant at p ≤ 0.05 and p ≤ 0.01, respectively.

**TABLE 4:** Effect of different cultivars and sulfur rates on plant height, spike length, branches per plant, spikes per plant, and spike weight of castor bean.

Cultivar	Sulfur rate (kg ha^–1^)	PH (cm)	SL (cm)	BPP	SPP	SDW (g)
NIAB-gold	0	98.50	34.33	3.50	5.24	125.4
	20	108.25	34.83	4.75	6.35	128.3
	40	110.76	35.13	5.25	6.97	136.7
	60	113.57	35.46	6.12	7.48	140.5
DS-30	0	110.87	32.2	4.12	5.17	115.5
	20	114.35	34.33	5.20	6.13	121.2
	40	116.78	34.7	5.85	6.56	136.4
	60	118.25	35.16	6.57	6.93	143.5
LSD at 5%		2.08	Ns	Ns	0.19	Ns
**Cultivar**						
NIAB-gold		107.77	34.94	4.90	6.51	132.72
DS-30		115.07	34.10	5.43	6.19	129.15
LSD at 5%		0.65	Ns	Ns	0.48	Ns
**Sulfur rates (kg ha^–1^)**						
0		104.69	33.26	3.81	5.20	120.45
20		111.30	34.58	4.97	6.24	124.75
40		113.77	34.91	5.55	6.76	136.55
60		115.91	35.31	6.34	7.20	142.00
LSD at 5%		1.47	Ns	0.12	0.13	4.97

*PH, plant height; SL, spike length; BPP, branches per plant; SPP, spike per plant; SDW, spike dry weight.*

By contrast, NIAB Gold produced higher spikes plant^–1^ than DS-30. Overall, maximum 7.2 spikes per plant were achieved with 60 kg S ha^–1^ fertilization, which was 38.4% more than with 0 kg S ha^–1^. It can be thought that S application improved plant N use efficiency, which affects the yield parameters such as plant height, branches, and spike plant^–1^ of castor bean. The total number of capsules depends on the length of the spike (SL). Data in Table 4 indicate that castor bean NIAB Gold maximum spike length (35.4 cm) was attained at 60 kg ha^–1^ S fertilization, which was increased non-significantly by 3.29% higher than control treatment and statistically similar to each other. DS–30 produced maximum spike length (35.16 cm) at 60 kg S ha^–1^, which was increased non-significantly by 9.2%, compared to control. The mean spike length of NIAB-Gold was 34.9 cm, which was statistically similar to DS–30. An average spike length of 35.3 cm was noted at 60 kg S ha^–1^, which increased non-significantly by 6.16%, compared to 0 kg S ha^–1^. Our findings are in confirmation with previous observations, showing that spike length increased with the application of S using gypsum or elemental S; however, molecular mechanisms behind improvement still need to be investigated (Chotchutima et al., 2016). Importantly, the molecular mechanism behind overall improvement of physio-morphological attributes is more crucial in sustainability perspectives (Javed et al., 2020).

Spike dry weight is the combination of seed, husk, and stalk. Cultivar NIAB Gold exhibited maximum spike dry weight (SDW) at 60 kg ha^–1^ S fertilization, which was 12% greater than that of the control (0 kg S ha^–1^) treatment and statistically similar to that of other treatments (Table 4). DS–30 produced significantly higher spike dry weight at 60 kg S ha^–1^, which was 24.24% higher than control. Data revealed that SDW was significantly (*p* ≤ 0.01) affected by S application. The increasing trend of SDW was observed with increasing S levels. The maximum SDW was achieved at 60 kg S ha^–1^, whereas lowest value was noticed when no S was applied. The mean spike dry weight of NIAB Gold was slightly higher as than that of DS–30. In the current study, an increasing trend in SDW was observed with the increasing S level. Fertilization of S enhances other nutrient uptake (especially N), which increased the biological yield of crops. Therefore, it can be assumed that the synergistic effect of S with nitrogen increased SDW of castor in the current study. Our results are in line with the previous study results, which showed that a higher level of S increased the biological yield of castor (Singh, 2003).

The sum of capsules plant^–1^ is the prime final yield participating factor. Castor bean NIAB Gold gave a maximum number of capsules (137) with the application of 60 kg S ha^–1^, which was 14.64% greater than that with the application of 0 kg S ha^–1^. Similarly, DS–30 produced comparatively more spike dry weight (306) at 60 kg S ha^–1^, which was 16.79% higher than that produced by control. Data revealed that both cultivars produced a comparable number of capsules plant^–1^. However, S levels significantly differed for the number of capsules per plant. The highest number of capsules was observed with 60 kg S, while the lowest value was observed in control. These results were comparable with Srivastava and Kumar (2015) results who observed a significantly increased capsule with the application of S using SSP, gypsum, and elemental S. A higher number of capsules were obtained with gypsum, followed by SSP.

### Days Taken to Flowering and Maturity

Castor bean has extended the duration of flowering because of its indeterminate growth. Both varieties have significant differences in 50% flowering, which was due to their genetic variability. Interestingly, S also has a significant (*p* ≤ 0.01) effect on days taken to maturity (Table 3). On an average, castor bean takes 78 days to attain 50% flowering with 60 kg S ha^–1^ fertilization, while control treatment takes 87 days. With respect to S levels, 0 kg S ha^–1^ accumulated higher growing degree days to attain 50% flowering (Table 5). These results are in agreement with Krishnamurthi and Mathan (1996) results which showed that fertilization at 45 kg S ha^–1^ decreased the duration taken to 50% flowering by about 3 days. Castor bean was harvested in two pickings based on the maturity of the main spikes and spikes that were formed on secondary branches. NIAB Gold takes 143 days to maturity, while DS-30 takes 181 days.

**TABLE 5:** Effect of different cultivars and sulfur rates on days taken to 50% flowering, capsule plant**^–^**^1^, hundred seed weight, days taken to maturity, and seed yield of castor bean.

Cultivar	Sulfur rate (kg ha^–1^)	DTF (days)	CPP	HSW (g)	DTM (days)	SY (Mg ha^–1^)
NIAB-gold	0	75.50	276	24.10	151.00	0.76
	20	71.75	279	24.34	143.50	0.86
	40	70.12	272	25.54	140.24	1.03
	60	69.15	317	26.25	138.30	1.18
DS-30	0	98.45	262	24.10	196.90	0.73
	20	89.13	275	24.57	178.26	0.84
	40	88.34	299	25.25	176.68	0.95
	60	87.87	306	25.80	175.74	1.12
LSD at 5%		2.69	Ns	Ns	5.37	Ns
**Cultivar**						
NIAB-gold		71.63	286.23	25.06	143.26	0.96
DS-30		90.94	285.75	24.93	181.90	0.91
LSD at 5%		11.65	Ns	Ns	23.33	Ns
**Sulfur rates (kg ha^–1^)**						
0		86.97	269	24.102	173.95	0.74
20		80.44	277	24.457	160.88	0.85
40		79.23	285	25.398	158.46	0.99
60		78.51	311	26.032	157.02	1.15
LSD at 5%		1.90	32.73	0.953	3.80	0.28

*DTF, days taken to 50% flowering; CPP, capsule per plant; HSW, hundred seed weight; SY, seed yield.*

### Seed Yield

Yield potential depends on the genetics of a crop, weather, and the nutrients that a crop takes from soil. Harvested seed yield is the most crucial attribute that determines the benefits from a crop in terms of output. NIAB Gold gave a maximum yield of 1.18 Mg ha^–1^ at 60 kg ha^–1^ S application, which was 54.86% higher than that of the control. A similar trend was noticed with DS–30 producing the highest seed yield (1.12 Mg ha^–1^) with 60 kg S ha^–1^, which was 52.78% higher than that of the control. The mean seed yield of NIAB Gold was 0.9626 Mg ha^–1^, which was statistically similar to that of the cultivar DS–30. ANOVA Table 3 shows that seed yield was significantly (*p* ≤ 0.01) influenced by S rates. On average, 60 kg S ha^–1^ produced markedly higher (54%) seed yield than the control (Table 5).

**FIGURE 3:**
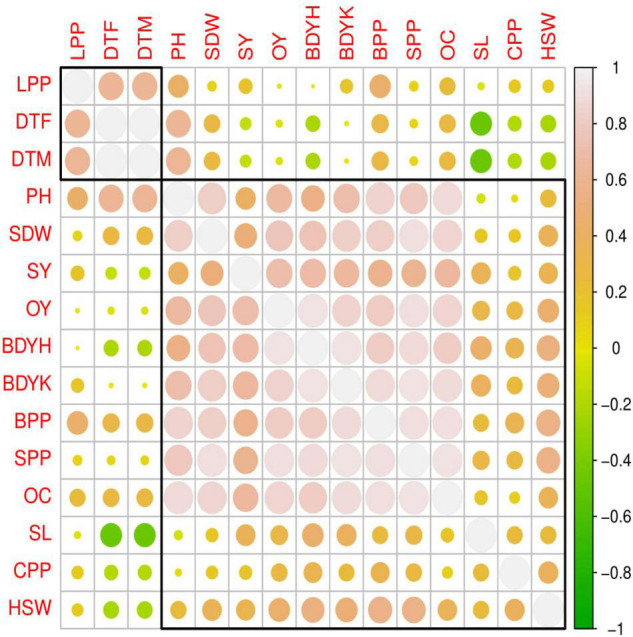
Pearson correlations among different parameters of caster bean studied in this experiment. Different abbreviations used in this figure are as follows: PH, plant height; SL, spike length; BPP, branches per plant; SPP, spike per plant; SDW, spike dry weight; DTF, days taken to 50% flowering; CPP, capsule per plant; HSW, hundred seed weight; SY, seed yield; DTM, days taken to maturity; OC, oil content; OY, oil yield; BDY, biodiesel yield.

Seed yield was strongly and positively associated with the branches and spikes per plant of castor bean (Figure 3). Srivastava and Kumar (2015) applied fertilization of S by various sources, and a seed yield of 2.27 Mg ha^–1^ was achieved at 30 kg ha^–1^ using gypsum. Anastasi et al. (2015) conducted a study on various cultivars of castor and found clear differences among cultivars for seed yield and attributes.

### Oil Content and Yield

The main purpose of growing oilseed crops is to get maximum oil. Among inedible oil seed crops, castor bean falls among top crops due to its high oil contents. Oil yield is the product of oil content (%) and seed yield. Data in Table 6 indicate that NIAB-Gold produced greatest oil yield (620.5 kg ha^–1^) in combination with fertilization of 60 kg S ha^–1^, while lowest oil yield (441 kg ha^–1^) was achieved at 0 kg S ha^–1^ from DS-30. NIAB-Gold had significantly higher (6.5%) oil yield than DS-30 fertilization. Overall mean oil yield was greatest at fertilization of 60 kg S ha^–1^, which was comparable to 40 kg S ha^–1^ (Table 6). NIAB Gold and DS–30 produced the maximum oil content at fertilization of 60 kg S ha^–1^. Maximum oil content of 56.4% was determined with NIAB Gold, while cultivar DS–30 gave a 54.4% oil content. Sulfur is an ingredient of coenzymes, vitamins, biotin, thiamine, and S-glycosides. The reason for higher oil contents and yield with increased level of S in the current study is that S plays a significant role for the production of chlorophyll and is the constituent of cystine, amino acids, methionine, and cystein (Chuah et al., 2016; Aguado-Deblas et al., 2020; Arslan et al., 2020). In the current study, oil yield was significantly and positively correlated with branches per plant, spike per plant, spike dry weight, oil yield, and oil contents of castor (Figure 3). A previous study also argued that S application improved oil yield and quality with increasing dose up to 75 kg ha^–1^ (Rehman and Farooq, 2013). In another study on castor bean, the highest oil contents (45.42%) were attained at 60 kg S ha^–1^ compared to 40, 20, and 0 kg S ha^–1^ (Zeinali et al., 2018).

**TABLE 6:** Effect of different cultivars and sulfur rates on oil content, oil yield, biodiesel yield kg^–1^ seed, and biodiesel yield ha^–1^ of castor bean.

Cultivar	Sulfur rate (kg ha^–1^)	OC (%)	OY (kg ha^1^)	BDY L kg^–1^	BDY L ha^–1^
NIAB-gold	0	42.8	422.63	0.303	300.07
	20	51.6	515.35	0.376	376.20
	40	52.8	569.22	0.396	426.91
	60	56.4	620.53	0.406	446.78
DS-30	0	46.4	437.52	0.255	240.64
	20	51.2	487.44	0.368	350.96
	40	53.7	516.95	0.397	382.54
	60	54.4	551.70	0.413	419.29
LSD at 5%		NS	NS	0.024	NS
**Cultivar**					
NIAB-gold		50.90	531.93	0.370	387.49
DS-30		51.42	498.40	0.358	348.36
LSD at 5%		NS	31.87	0.10	17.96
**Sulfur rates (kg ha^–1^)**					
0		44.60	430.08	0.279	270.35
20		51.40	501.39	0.372	363.58
40		53.25	543.08	0.396	404.73
60		55.40	586.11	0.409	433.04
LSD at 5%		2.36	47.36	0.17	33.72

*DTM, days taken to maturity; OC, oil content; OY, oil yield; BDY, biodiesel yield.*

### Biodiesel Production

The main aim of this study was to enhance biodiesel yield (BDY) on a hectare basis. BDY from both cultivars increased significantly with S application. BDY increased 34% per kilogram of seed with changing S dose from 0 to 60 kg S ha^–1^ (Table 6). As BDY increased from seed it also increased in hectares, from NIAB Gold maximum 446.78 L ha^–1^ biodiesel obtained with 60 kg S ha^–1^ and lowest 300.07 L ha^–1^ at 0 k S ha^–1^. From DS-30 cultivar, minimum 240.64 L ha^–1^ biodiesel was obtained with 0 kg S ha^–1^ and maximum 419.29 L ha^–1^ was obtained at application of 60 kg S ha^–1^. Biodiesel yield per hectare was the product of oil yield per hectare and oil contents. Due to higher oil yield per hectare and oil content, NIAB Gold gave higher BDY per hectare. Similarly, the increasing trend of S clearly enhanced BDY per hectare of castor bean (Table 6).

Strongly positive and significant correlation of BDY per hectare was observed with branches per plant, spikes per plant, spike weight, oil contents, oil yield, and seed yield (Figure 3). There are many factors that significantly affect the BDY such as the methanol-to-oil ratio, temperature, catalyst dose, stirring speed, and reaction time. As a result of aforementioned conditions, castor bean oil depicted a maximum 76% biodiesel yield. Experimental results revealed that higher ratios of methanol to oil results in greater meta-analysis and consequently yielded higher biodiesel. However, further increase in methanol causes difficulty during product separation after chemical reaction, which leads to lower FAME yield (Saleem et al., 2020b). The yield of biodiesel obtained from castor bean oil was 71–75% in NIAB Gold cultivar and 55–76% in DS–30 (Table 7). The observations are in line with the findings of Naseem et al. (2019) who obtained 75% biodiesel from castor bean. Thirumarimurugan et al. (2012) obtained 80% biodiesel yield after transesterification of oil seed crop.

**TABLE 7:** Effect of sulfur and cultivar on oil and biodiesel quality parameters of castor bean.

	Castor oil parameters					Castor biodiesel parameters							
Treatments	Oil yield (%)	acid value (%)	Viscosity at 25 °C (mm^2^ s^–1^)		Yield (%) at 5:1 m/o	AV mg KOH/g	V mm^2^ s^–1^	SV mg KOH/g	IV g I^2^/100g	CV	CN	CP °C	PP °C

				**ASTM standards**		**<0.5**	**1.9–6.0**	**<312**	**<120**	**kJ/g**	**47 ≤**	**−3 to 12**	**−15 to 10**
NG S_1_	52.8	1.68	272.2		71	0.56	23.67	207.57	95.08	41.2	51.20	−1	−7
NG S_2_	51.6	1.68	238.5		73	0.56	18.22	221.60	89.60	41.5	50.77	−2	−9
NG S_3_	42.8	1.12	281.6		75	0.56	19.89	218.79	92.03	43.7	50.54	−2	−6
NG S_4_	56.4	1.68	220.0		72	0.56	17.22	224.40	87.16	42.1	51.01	−2	−7
DS S_1_	51.2	2.24	401.8		55	1.12	18.56	187.94	99.96	41.1	52.85	−3	−5
DS S_2_	46.4	1.68	406.9		72	0.56	23.78	232.82	102.40	41.7	46.70	−1	−4
DS S_3_	53.7	1.68	451.4		74	0.56	25.00	193.55	97.52	43.9	52.56	−1	−5
DS S_4_	54.4	1.68	207.5		76	0.56	25.89	187.94	103.62	42.5	52.03	−3	−6
Mustard oil (Shahzadi et al., 018)	–	–	–		–	0.37	5.8	224	81.8	42.9	52	−1	−12
Waste cooking oil (Ghani et al., 2020)	–	–	—		–	0.5	5.88	286	59	37.8	52	1	−7
Soapnut oil Biodiesel (Ghani et al., 2021)	–	–	–		–	0.5	5.8	226	29	36	63	6	−2

*AV, acid value; V, viscosity; SV, saponification value; IV, iodine value; CV, calorific value; CP, cloud point; PP, pure point.*

### Quality of Synthesized Biodiesel

After the conversion of different oil samples into biodiesel, the physio-chemical properties were also determined. The efficiency of biodiesel as a fuel is determined by a very important property known as kinematic viscosity. The resistance in flow of fluids is directly related to viscosity (Salaheldeen et al., 2015; Saleem et al., 2020b). Under cold conditions, high viscosity is not suitable because as temperature decreases, the viscosity of fuel increases (Chotchutima et al., 2016). According to ASTM standards, the kinematic viscosity BD should be between 1.9 and 6 mm^2^ s^–1^. The viscosity of castor bean oil and their synthesized methyl ester was checked, and it was seen that for castor bean oil, viscosity ranged between 220 and 281.6 mm^2^ s^–1^ in NIAB Gold and 207.5–451.4 mm^2^ s^–1^ in DS–30. As a result of transesterification, the viscosity of oil was significantly reduced. The viscosity of biodiesel ranged from 17.22 to 23.67 mm^2^ s^–1^ and from 18.56 to 25.89 mm^2^ s^–1^ in NIAB Gold and DS-30, respectively (Table 7). The kinematic viscosity of castor bean biodiesel is higher than ASTM standard, which might be due to the existence of hydroxyl groups (Okullo et al., 2012). According to a study reported by Tunio et al. (2016), the viscosity of castor bean biodiesel and oil was found to be 7.5 mm^2^ s^–1^ and 196 mm^2^ s^–1^, respectively. Okullo et al. (2012) found that the viscosity of castor bean biodiesel was 10.75 mm^2^ s^–1^.

The observed values of biodiesel ranged from 41.2 to 43.7 kJ g^–1^and from 41.1 to 43.9 kJ g^–1^ for NIAB Gold and DS–30, respectively. The calorific value of castor bean was reported to be 38.4 kJ g^–1^ (Keera et al., 2018). The observed values from castor bean biodiesel were in the ASTM range. Generally, the calorific value of biodiesel is lower than that of the petro-diesel, but in biodiesel, due to the higher amount of oxygen, complete combustion is possible in the engine (Ramadhas et al., 2005). Acid value ranged from 1.12 to 1.68% in NIAB Gold and from 1.68 to 2.24% in the cultivar DS–30 (Table 7). As a result of transesterification, the acid value of oil was significantly reduced. The acid value of biodiesel was found to be 0.56 mg NaOH g–1 and 0.56–1.12 mg NaOH g^–1^ for NIAB Gold and DS-30, respectively. Okullo et al. (2012) reported the castor bean biodiesel acid value was 0.35 mg NaOH g^–1^. The mass of iodine measured in grams present in 100 g of given oil is known as the iodine value. Iodine value is often used to measure the amount of unsaturation fatty acids in oil (Knothe, 2006). The vodine value of synthesized biodiesel for NIAB Gold was found in the range of 87.16–95.08 g I2/100 g and 97.52–103.62 g I2/100 g for DS–30. The iodine values in the present study are close to the observations of other researchers in castor bean (Bauddh and Singh, 2012). The lower the saponification, the higher the BDY because higher value led to soap formation (Keera et al., 2018; Osorio-González et al., 2020). According to ASTM standards, the iodine value should be less than 312 mg NaOH g^–1^. In the present study, saponification of synthesized biodiesel from castor bean NIAB Gold was found to be 207.57–224.40 mg NaOH g^–1^, and for DS–30, it was 187.94–232.82 mg NaOH g^–1^ (Table 7). According to Ferdous et al. (2012), the saponification value of castor bean biodiesel was reported in mg KOH g^–1^.

The cetane number is a very important fuel property used to check the tendency of the fuel to ignite spontaneously. The cetane number of diesel mainly depends on the carbon number, parent ester concentration, and molecular structure (Murphy et al., 2004). The recommended range of the cetane number for biodiesel according to the ASTM standard should be from 46 to 52, and for petroleum diesel, the range of the cetane number can be from 40 to 55 (Shahzadi et al., 2018). The cetane number of synthesized castor bean biodiesel from NIAB Gold was found for DS–30 was checked by using standard method described in section “Determination of Cetane Number” (Naseem et al., 2019), and it was found to be 46.70–52.85 (Table 7). These results were within ASTM standard limits. Our results are similar to those of Conceicao et al. (2007) who reported a cetane number of 50 in biodiesel obtained from castor bean. The cloud point of synthesized biodiesel obtained from castor bean oil was found in the range of −1 to −3°C. The reported value of cloud point for castor bean biodiesel methyl ester was 3°C (Conceicao et al., 2007). The pour point primarily depends on the structure of oil and feedstock used for biodiesel production. In the current study, the pour point of produced biodiesel from castor oil was found to be −9°C (DS-30) (Table 7). The pour point value observed in our trial is lower than that in previous observation, which reported the value of pure point for castor bean biodiesel methyl ester was 6°C (Ferdous et al., 2012). This might be due to the differences in both studies.

### Principal Component Analysis

**FIGURE 4:**
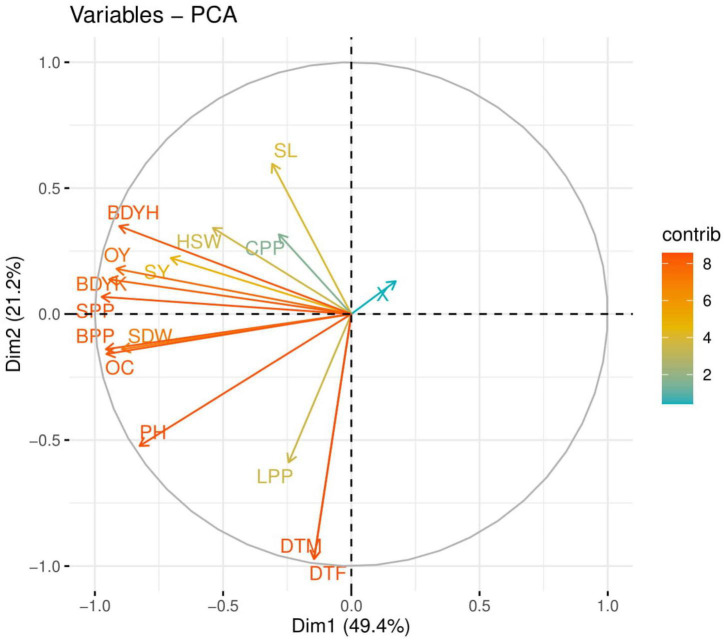
Loading plots of principal component analysis (PCA) on different studied attributes of caster bean varieties under different fertilizations of S in the soil. Different abbreviations used in this figure are as follows: PH, plant height; SL, spike length; BPP, branches per plant; SPP, spike per plant; SDW, spike dry weight; DTF, days taken to 50% flowering; CPP, capsule per plant; HSW, hundred seed weight; SY, seed yield; DTM, days taken to maturity; OC, oil content; OY, oil yield; BDY, biodiesel yield.

The loading plots of PCA to evaluate the effect of fertilization of S on both cultivars of coater beans (NIAB-Gold and DS-30) are presented in Figure 4. Of all the main components, the first two components—Dim1 and Dim2—comprise more than 70.6% of the whole database and make up the largest portion of all components (Figure 4). Among this, Dim1 contributes 49.4%, and Dim2 contributes 21.2% of the whole dataset. However, all studied parameters were negatively correlated in the database with each other.

## Conclusion

Castor bean has a huge potential as a non-conventional energy source to supply biodiesel. In this study, genetic variability in castor bean varieties influenced the growth and yield-related characteristics, whereas S had a remarkable influence on seed yield and oil content. The higher level of S application had an advantage over lower levels concerning seed and oil yield and oil content of castor bean. In this view, both the tested cultivars produced the highest seed yields, oil contents, and biodiesel yield at 60 kg S ha^–1^. The results also reveal that cultivar NIAB Gold has the advantage of producing greater seed yield (6% more) than DS-30. All quality parameters (except BD viscosity) of castor bean biodiesel were in the ASTM standard range. However, higher BD viscosity than ASTM standards was due to the high viscosity of castor bean oil.

## Data Availability Statement

The raw data supporting the conclusions of this article will be made available by the authors, without undue reservation.

## Author Contributions

MA and SS: conceptualization. AMa: methodology. AMu: software, formal analysis, investigation, and writing—original draft preparation. TJ and RS: validation. MA: resources, supervision, and project administration. MA, AMa, and TJ: data curation. TJ, AMu, DS, AAS, RA, ANS, RS, SS, and SSA: writing—review and editing. All authors have read and agreed to the published version of the manuscript.

## Conflict of Interest

The authors declare that the research was conducted in the absence of any commercial or financial relationships that could be construed as a potential conflict of interest.

## Publisher’s Note

All claims expressed in this article are solely those of the authors and do not necessarily represent those of their affiliated organizations, or those of the publisher, the editors and the reviewers. Any product that may be evaluated in this article, or claim that may be made by its manufacturer, is not guaranteed or endorsed by the publisher.

## References

[B1] Aguado-Deblas L. Hidalgo-Carrillo J. Bautista F. M. Luna D. Luna C. Calero J. (2020). Acetone prospect as an additive to allow the use of castor and sunflower oils as drop-in biofuels in diesel/acetone/vegetable oil triple blends for application in diesel engines. *Molecules* 25:2935. 10.3390/molecules25122935 32630602 PMC7356534

[B2] Ahmad G. Jan A. Arif M. Jan M. Khattak R. (2007). Influence of nitrogen and sulfur fertilization on quality of canola (*Brassica napus* L.) under rainfed conditions. *J. Zhejiang Univ. Sci. B* 8 731–737. 10.1631/jzus.2007.B0731 17910116 PMC1997227

[B3] Anastasi U. Sortino O. Cosentino S. Patanè C. (2015). Seed yield and oil quality of perennial castor bean in a mediterranean envi-ronment. *Int. J. Plant Prod.* 9 99–116.

[B4] Arslan A. Rancke-Madsen A. Brask J. (2020). Enzymatic synthesis of estolides from castor oil. *Catalysts* 10:835. 10.3390/catal10080835

[B5] Azam A. Shafique M. (2017). Agriculture in Pakistan and its impact on economy. *A Review. Inter. J. Adv. Sci. Technol.* 103 47–60. 10.14257/ijast.2017.103.05

[B6] Bauddh K. Singh R. P. (2012). Growth, tolerance efficiency and phytoremediation potential of *Ricinus communis* (L.) and *Brassica juncea* (L.) in salinity and drought affected cadmium contaminated soil. *Ecotoxicol. Environ. Saf.* 85 13–22. 10.1016/j.ecoenv.2012.08.019 22959315

[B7] Burt B. G. Mealy W. C. (1942). *Process of Making Pure Soaps. Google Patents US2271619A.* Washington, DC: United States Government Publishing Office.

[B8] Carvalho M. Torres F. Ferreira V. Silva J. Martins J. Torres E. (2020). Effects of diethyl ether introduction in emissions and per-formance of a diesel engine fueled with biodiesel-ethanol blends. *Energies* 13:3787. 10.3390/en13153787

[B9] Cheema N. M. Farooq U. Shabbir G. Shah M. K. N. Musa M. (2013). Prospects of castor bean cultivation in rain fed tract of Pakistan. *Pak J. Bot.* 45 219–224.

[B10] Chen R. Blagodatskaya E. Senbayram M. Blagodatsky S. Myachina O. Dittert K. (2012). Decomposition of biogas residues in soil and their effects on microbial growth kinetics and enzyme activities. *Biomass Bioenergy* 45 221–229. 10.1016/j.biombioe.2012.06.014

[B11] Chotchutima S. Tudsri S. Kangvansaichol K. Sripichitt P. (2016). Effects of sulfur and phosphorus application on the growth, bio-mass yield and fuel properties of leucaena (*Leucaena leucocephala* (Lam.) de Wit.) as bioenergy crop on sandy infertile soil. *Agric. Nat. Resour.* 50 54–59. 10.1016/j.anres.2015.09.002

[B12] Chuah L. F. Yusup S. Aziz A. R. A. Klemeš J. J. Bokhari A. Abdullah M. Z. (2016). Influence of fatty acids content in non-edible oil for biodiesel properties. *Clean Technol. Environ. Policy* 18 473–482. 10.1007/s10098-015-1022-x

[B13] Conceicao M. M. Candeia R. A. Silva F. C. Bezerra A. F. Fernandes V. J. Jr. Souza A. G. (2007). Thermoanalytical characterization of castor oil biodiesel. *Renewable Sust. Energy Rev.* 11 964–975. 10.1016/j.rser.2005.10.001

[B14] Ferdous K. Uddin M. R. Deb A. Ferdous J. Khan M. R. Islam M. (2012). Preparation of biodiesel from castor oil by two-step method. *SUST J. Sci. Technol.* 20, 48–56.

[B15] Ghani N. Iqbal J. Sadaf S. Bhatti H. N. Asgher M. (2020). Comparison of photo-esterification capability of bismuth vanadate with reduced graphene oxide bismuth vanadate (RGO/BiVO_4_) composite for biodiesel production from high free fatty acid containing non-edible oil. *Chemistryselect* 5 9245–9253. 10.1002/slct.202001913

[B16] Ghani N. Iqbal J. Sadaf S. Bhatti H. N. Asgher M. (2021). A facile approach for the synthesis of SrTiO_3_/g-C_3_N_4_ Photo-catalyst and its efficacy in biodiesel production. *Chemistryselect* 6 12082–12093. 10.1002/slct.202101787

[B17] Hashem I. A. Abbas A. Y. Abd El-Hamed A. E.-N. H. Salem H. M. S. El-hosseiny O. E. M. Abdel-Salam M. A. (2020). Potential of rice straw biochar, sulfur and ryegrass (*Lolium perenne* L.) in remediating soil contaminated with nickel through irrigation with untreated wastewater. *PeerJ* 8:e9267. 10.7717/peerj.9267 32566397 PMC7295020

[B18] Javed T. Ali M. M. Shabbir R. Anwar R. Afzal I. Mauro R. P. (2021). Alleviation of copper-induced stress in pea (*Pisum sativum* L.) through foliar application of gibberellic acid. *Biology* 10:120. 10.3390/biology10020120 33562436 PMC7915894

[B19] Javed T. Shabbir R. Ali A. Afzal I. Zaheer U. Gao S. J. (2020). Transcription factors in plant stress responses: challenges and poten-tial for sugarcane improvement. *Plants* 9:491. 10.3390/plants9040491 32290272 PMC7238037

[B20] Kamran M. Parveen A. Ahmar S. Malik Z. Hussain S. Chattha M. S. (2019). An overview of hazardous impacts of soil salinity in crops, tolerance mechanisms, and amelioration through selenium supplemen-tation. *Int. J. Mol. Sci.* 21:148. 10.3390/ijms21010148 31878296 PMC6981449

[B21] Keera S. El Sabagh S. Taman A. (2018). Castor oil biodiesel production and optimization. *Egypt. J. Pet.* 27 979–984. 10.1016/j.ejpe.2018.02.007

[B22] Knothe G. (2006). Analyzing biodiesel: standards and other methods. *J. Am. Oil Chem. Soc.* 83 823–833. 10.1007/s11746-006-5033-y

[B23] Krishnamurthi V. Mathan K. (1996). Influence of sulphur and magnesium on growth and yield of sunflower (*Helianthus annuus*). *Indian J. Agron.* 41 627–629.

[B24] Lingaiah V. Rajasekaran P. (1986). Biodigestion of cowdung and organic wastes mixed with oil cake in relation to energy. *Agric. Wastes* 17 161–173. 10.1016/0141-4607(86)90091-0

[B25] Mahmood A. Awan M. I. Sadaf S. Mukhtar A. Wang X. Fiaz S. (2021). Bio-diesel production of sunflower through sulphur management in a semi-arid subtropical environment. *Environ. Sci. Pol. Res.* 29 13268–13278. 10.1007/s11356-021-16688-z 34585347

[B26] Martins A. E. Pereira M. S. Jorgetto A. O. Martines M. A. Silva R. I. Saeki M. J. (2013). The reactive surface of castor leaf [*Ricinus communis* L.] powder as a green adsorbent for the removal of heavy metals from natural river water. *Appl. Surf. Sci.* 276 24–30. 10.1016/j.apsusc.2013.02.096

[B27] Muanruksa P. Dujjanutat P. Kaewkannetra P. (2020). Entrapping immobilisation of lipase on biocomposite hydrogels toward for biodiesel production from waste frying acid oil. *Catalysts* 10:834. 10.3390/catal10080834

[B28] Murphy M. J. Taylor J. D. McCormick R. L. (2004). *Compendium of Experimental Cetane Number Data.* Golden, CO: National Renewable Energy Labor-atory. 10.2172/1086353

[B29] Mutlu H. Meier M. A. (2010). Castor oil as a renewable resource for the chemical industry. *Eur. J. Lipid Sci. Technol.* 112 10–30. 10.1002/ejlt.200900138

[B30] Narayanamma V. (2018). Potentiality of ericulture for poverty alleviation in dry land areas of Telangana-an economic analysis. *J. Appl. Zool. Res.* 29 161–168.

[B31] Naseem M. Sadaf S. Bibi S. Aziz S. Ullah I. (2019). Evaluation of a NIAB gold castor variety for biodiesel production and bio-pesticide. *Indus. Crops Prod.* 130 634–641. 10.1016/j.indcrop.2019.01.022

[B32] Novaes M. A. S. Veloso C. M. Siqueira O. H. G. B. D. Ferreira M. F. L. Lovatti J. V. R. Oliveira H. R. (2020). Use of castor bean meal, biodiesel industry coproduct, in a lamb production system using creep-feeding in Brazil. *Animals* 10:1250. 10.3390/ani10081250 32717900 PMC7459915

[B33] Okullo A. A. Temu A. Ogwok P. Ntalikwa J. (2012). Physico-chemical properties of biodiesel from jatropha and castor oils. *Int. J. Renew. Energy Res.* 2 47–52.

[B34] Oliveira L. B. de Araujo M. S. M. Rosa L. P. Barata M. La Rovere E. L. (2008). Analysis of the sustainability of using wastes in the Brazilian power industry. *Renew. Sust. Energy Rev.* 12 883–890. 10.1016/j.rser.2006.10.013

[B35] Osorio-González C. S. Gómez-Falcon N. Sandoval-Salas F. Saini R. Brar S. K. Ramírez A. A. (2020). Production of biodiesel from castor oil: a review. *Energies* 13:2467. 10.3390/en13102467 30225073

[B36] Ramadhas A. S. Jayaraj S. Muraleedharan C. (2005). Biodiesel production from high FFA rubber seed oil. *Fuel* 84 335–340. 10.1016/j.fuel.2004.09.016

[B37] Ramanjaneyulu A. Anudradha G. Ramana M. V. Reddy A. Gopal N. M. (2017). Multifarious uses of castor (*Ricinus communis* L.). *Int. J. Econ. Plants* 4 170–176.

[B38] Ramanjaneyulu A. Vishnuvardhan Reddy A. Nagesh Kumar M. Gouri Shankar V. Dharma Reddy K. (2013). Development and dif-fusion of rabi castor technology. *Tech. Bull.* 1 1–30.

[B39] Rehman A.-U. Farooq M. (2013). Boron application through seed coating improves the water relations, panicle fertility, kernel yield, and biofortification of fine grain aromatic rice. *Acta Physiol. Plant.* 35 411–418. 10.1007/s11738-012-1083-y

[B40] Ren C. You J. Qi Y. Huang G. Hu H. (2017). Effects of sulfur on toxicity and bioavailability of Cu for castor (*Ricinus communis* L.) in Cu-contaminated soil. *Environ. Sci. Pollut. Res.* 24 27476–27483. 10.1007/s11356-017-0306-6 28980167

[B41] Salaheldeen M. Aroua M. K. Mariod A. Cheng S. F. Abdelrahman M. A. Atabani A. (2015). Physicochemical characterization and thermal behavior of biodiesel and biodiesel–diesel blends derived from crude *Moringa peregrina* seed oil. *Energy Conver. Manag.* 92 535–542. 10.1016/j.enconman.2014.12.087

[B42] Saleem M. H. Ali S. Rehman M. Rana M. Rizwan M. Kamran M. (2020a). Influ-ence of phosphorus on copper phytoextraction via modulating cellular organelles in two jute (*Corchorus capsularis* L.) varieties grown in a copper mining soil of Hubei Province, China. *Chemosphere* 248:126032. 10.1016/j.chemosphere.2020.126032 32018110

[B43] Saleem M. H. Rehman M. Kamran M. Afzal J. Noushahi H. A. Liu L. (2020b). Investigating the potential of different jute varieties for phytoremediation of copper-contaminated soil. *Environ. Sci. Polluti. Res.* 27 30367–30377. 10.1007/s11356-020-09232-y 32462620

[B44] Shahzadi I. Sadaf S. Iqbal J. Ullah I. Bhatti H. N. (2018). Evaluation of mustard oil for the synthesis of biodiesel: pretreatment and optimization study. *Environ. Prog. Sust. Energy* 37 1829–1835. 10.1002/ep.12833

[B45] Sharma H. Gupta A. (2003). Effect of sulphur on growth parameters and yield of some selected crops. *Ann. Agric. Res.* 24 136–138. 12941977

[B46] Singh I. (2003). Studies on effect of spacing in castor (*Ricinus communis* L.). *Ann. Arid Zone* 42 89–92.

[B47] Srivastava S. Kumar J. (2015). Response of castor (*Ricinus communis* L.) to sulphur under irrigated conditions of Uttar Pradesh. India. *Plant Arch.* 15 879–881.

[B48] Steel R. G. (1997). *Pinciples and Procedures of Statistics a Biometrical Approach.* New York, NY: McGraw-Hil, 0070610282.

[B49] Sutton W. J. Bozzo G. G. Carlow C. MacDonald W. N. Shelp B. J. (2019). Strategic timing and rate of sulphur fertilization improves sulphur use efficiency in subirrigated greenhouse-grown chrysanthemums. *Can. J. Plant Sci.* 99 654–665. 10.1139/cjps-2018-0334

[B50] Thirumarimurugan M. Sivakumar V. Xavier A. M. Prabhakaran D. Kannadasan T. (2012). Preparation of biodiesel from sunflower oil by transesterification. *Int. J. Biosci. Bioch. Bioinform.* 2:441. 10.7763/IJBBB.2012.V2.151

[B51] Toldrá-Reig F. Mora L. Toldrá F. (2020). Developments in the use of lipase transesterification for biodiesel production from animal fat waste. *Appl. Sci.* 10:5085. 10.3390/app10155085

[B52] Tunio M. Samo S. R. Ali Z. M. Jakhrani A. Mukwana K. (2016). Production and characterization of biodiesel from indigenous castor seeds. *Int. J. Eng. Appl. Sci.* 3 28–33.

[B53] Vwioko D. Anoliefo G. Fashemi S. (2006). Metal concentration in plant tissues of *Ricinus communis* L. (castor oil) grown in soil con-taminated with spent lubricating oil. *J. Appl. Sci. Environ. Manag.* 10 127–134. 10.4314/jasem.v10i3.17331

[B54] Zaheer I. E. Ali S. Saleem M. H. Imran M. Alnusairi G. S. H. Alharbi B. M. (2020). Role of iron–lysine on morpho-physiological traits and combating chromium toxicity in rapeseed (*Brassica napus* L.) plants irri-gated with different levels of tannery wastewater. *Plant Physiol. Biochem.* 155 70–84. 10.1016/j.plaphy.2020.07.034 32745932

[B55] Zahir A. A. Rahuman A. A. Bagavan A. Santhoshkumar T. Mohamed R. R. Kamaraj C. (2010). Evaluation of botanical extracts against Haemaphysalis bispinosa Neumann and Hippobosca maculata Leach. *Parasitol. Res.* 107 585–592. 10.1007/s00436-010-1898-7 20467752

[B56] Zeinali A. Sadeghi B. A. R. Sarabi V. (2018). Investigation of nitrogen and sulphur effects on quantitative and qualitative characteris-tics of castor bean seed (*Ricinus communis* L.). *Ira. J. Field Crop Sci.* 49 29–43.

